# Textual complexity adjustments to the English reading comprehension test for undergraduate EFL students

**DOI:** 10.1016/j.heliyon.2023.e12891

**Published:** 2023-01-10

**Authors:** Helta Anggia, Anita Habók

**Affiliations:** a Doctoral School of Education, University of Szeged, Hungary; b Institute of Education, University of Szeged, MTA-SZTE Digital Learning Technologies Research Group, Hungary

**Keywords:** Textual complexity, Text difficulty level, Reading comprehension test, Text variables, Reader variables

## Abstract

It can be challenging for teachers to prepare students for a reading comprehension test. While most research on reading comprehension tests focuses on the interaction between the text complexity and test-taker's ability, this study investigates the interaction between the text complexity and degree of difficulty of the tasks, following each text in an adapted reading comprehension test. The experiment examined the plausibility of adapting a reading comprehension test for university students through textual complexity management. It involved undergraduate English-*as*-a-foreign-language (EFL) participants (N = 1000) with English proficiency levels ranging from A1 to C2. A 38-item reading comprehension test with textual complexity adjustment was adapted. Item fit was assessed using the Rasch model analysis. ANOVA was performed to determine which reading comprehension subsamples differed significantly, whether the difficulty level of the test confirmed that of the texts, and what cognitive process contributed the most to test difficulty. The findings generated 32 qualified items, which fit the Rasch model. Participants significantly differed in reading comprehension, indicating the test's ability to differentiate the participants based on their classification. The study carried an implication that the difficulty of reading comprehension test is not solely contingent on textual complexity but also relies on task difficulty. Therefore, teachers must pay attention to both when preparing students for a reading comprehension test.

## 1 Introduction

A reading comprehension tester must consider the important role of the test-taker and text aspects when developing a reading comprehension test [1]. Most evaluations of the psychometric properties of reading comprehension tests combine these two variables to determine a test's optimal fitness [2,3]. It highlights the importance of matching the characteristics of EFL readers with the text difficulty level of the reading comprehension test. EFL readers' characteristics include linguistic competence, schemata, cultural knowledge, world knowledge, interests, reading goals, reading strategies, metacognitive awareness [[4], [5], [6], [7], and metalinguistics [8].

The essential characteristics supporting EFL readers' reading comprehension include their metacognitive awareness in reading [9]. Metacognitive awareness refers to one's ability to control their cognitive skills in learning. A study of 302 native English and English-*as*-a-second-language (ESL) students in a U.S. college found that when reading academic content, native students used more cognitive and metacognitive skills than their ESL counterparts [10]. As native readers, they have no concerns regarding linguistic knowledge and can therefore control their cognitive skills better than ESL/EFL students when reading.

Meanwhile, adult EFL readers, despite being relatively mature and possessing knowledge of the world concept, may lack linguistic knowledge, which may then occasionally delay their comprehension and leave them little opportunity to improve their cognitive and metacognitive skills [11], a condition that undergraduate EFL students in Indonesia might encounter. However, no studies have clearly described how testers prepare, develop, or adopt reading comprehension tests for university students in Indonesia, especially during their participation in mandatory English programs held by their respective universities. More often than not, instructors assess students' English proficiency, including reading comprehension, merely for the sake of examination without considering students' scores to strategize future assessment. As a result, no account has been provided as to whether the texts or passages in the tests that students read and take match their linguistic ability. This problem arises from teachers' inability to fit the appropriate text difficulty in a reading test with students’ linguistic skills. This study is an attempt to address and approach this issue.

When selecting the text difficulty level of reading tests, testers must address the gap between EFL and native readers in terms of linguistic knowledge and metacognitive awareness [12,13]. Linguistic knowledge is the most important aspect for EFL readers to understand English texts, followed by metacognitive awareness in reading [14]. A reading comprehension tester must consider determinant factors such as EFL readers’ individual differences in linguistic knowledge when developing or adapting appropriate reading comprehension tests.

Meanwhile, essential text variables include readability, genre, text topic and content, linguistic factors, and others [15]. The readability of English texts is especially crucial for the evaluation of EFL students’ reading comprehension skills. Barrot [16]and Reed and Kershaw-Herrera [17] considered textual complexity in preparing their respective reading comprehension tests, concluding that text readability, particularly in longer paragraphs and passages, was a dominant factor that influences reading comprehension, especially among adolescents and adult EFL readers. The current study used an adapted reading comprehension test with textual complexity management to match test-takers with the appropriate text difficulty according to their reading level. This research also investigated how text difficulty and reading comprehension subskills influence test difficulty.

The study findings should lead to some important insights; first, fitting the items to the Rasch model guaranteed the fitness of the items to the students’ ability. Second, the answer to the research question will help reveal whether the task difficulty levels are contingent on the textual complexity of the texts in the reading test. This information can help instructors understand which aspects of the test are challenging for the students. Third, the study should inform educators what reading tasks students need to anticipate in order to perform well on the reading test, according to its difficulty level. This will enable both the instructor and students to focus on appropriate strategies. Fourth, the findings are expected to confirm, strengthen, and add to the current theory of textual complexity comprising the integration of text complexity, task, and readers. If any result contradicts the existing theories of textual complexity, it could be an addition to those theories. Finally, language teachers, particularly reading comprehension instructors, will benefit the most from the findings of this study since they can replicate the method of textual complexity adjustment to determine test difficulty levels.

## 2 Theoretical background

### 2.1 Text complexity and text difficulty

**Fig. 1:**
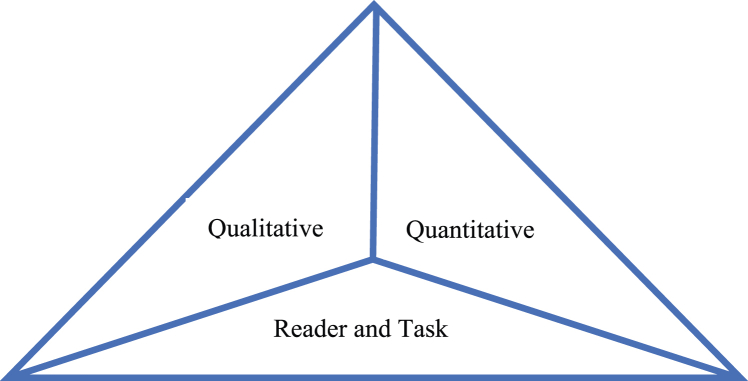
Three-part model for text complexity.

Shanahan [19] defines textual complexity as a characteristic of the text and applies a three-part model that includes the reader and text as well as qualitative and quantitative measures (see Fig. 1). The quantitative measure assesses a text's complexity by taking into account word and sentence length, word occurrence, and Lexile levels, whereas the qualitative measure relates to making an informed judgment of a text's difficulty. Additionally, a reader and text match refers to the instructor's attempt to balance the difficulty of the text with the reading level of the student.

**Fig. 2:**
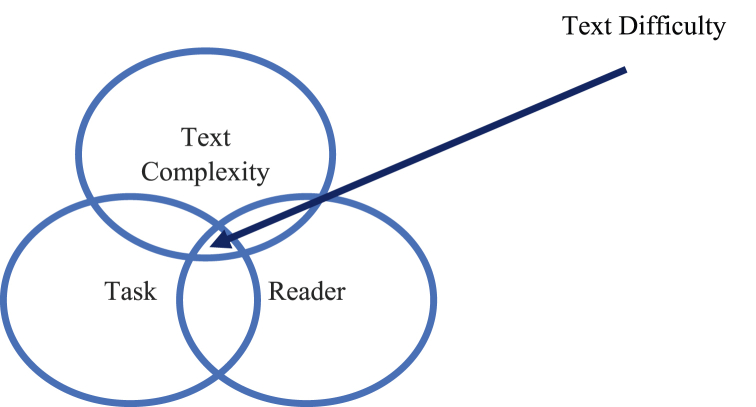
Heuristic comprehension model.

Mesmer et al. [20] distinguish text complexity from text difficulty due to the terms’ frequent use as synonyms. Text complexity is based on the measured aspects that Shanahan outlined, but text difficulty occurs as a result of the interaction of the text complexity, reader, and task. In line with Mesmer et al. [20], Amendum et al. [1] based their definition of text difficulty on the comprehension heuristic by RAND [21] that put text difficulty as an accumulating measurement resulting from the interaction between text complexity, reader, and task (see Fig. 2).

As mentioned, this study is based on the theory of text complexity and difficulty. Using the three-part model, we employed at least the quantitative measure of the texts in the reading test and matched the text complexity with the students’ reading ability. As the reading test had been prepared, we checked whether the difficulty level of the reading tasks following each reading text matched with the text complexity. Here the heuristic comprehension model helped explain the reading test difficulty level as we tested the students and observed the interaction between the three aspects of the model.

### 2.2 Reader variables: EFL readers’ metacognitive awareness and cognitive process

Nothing is more critical for EFL readers than metacognitive awareness and cognitive processes that facilitate learning and problem-solving in reading [22]. Metacognitive awareness, or metacomprehension, refers to native readers’ core ability that allows them to be aware of their reading comprehension level and manage their learning to reach a certain degree of comprehension [23]. It makes sense that the metacognitive awareness of EFL readers tends to be lower than that of native readers, as EFL readers initially struggle with linguistic knowledge before developing their metacognitive awareness [24]. In a sense, metacognitive awareness is closely associated with readers who display high ability either in comprehension or language proficiency since they already have sufficient capital to mediate the necessary cognitive processes for solving reading assignments [25,26].

What seems common among EFL readers in their metacognitive awareness when dealing with reading text difficulty is that they use problem-solving strategies more frequently than global and supportive strategies in their efforts to understand foreign-language text with significant linguistic difficulty [23,27]. This is clearly because EFL readers often encounter challenges in processing text with low readability, unfamiliar topic and content, and a variety of genres as previously suggested by Alderson [28], Barrot [16] Oakland and Lane [18], Reed and Kershaw-Herrera [17]. Notably, readers demonstrate their problem-solving skills when confronted with a text rich in lexical diversity, rereading the text, slowing their pace, and concentrating more intently [23,25].

In general, cognitive processes refer to the transfer of information to connect various inputs and set a cognitive system to stimulate a response and behavioral output; these processes typically involve perception, memory, learning, emotion, intentionality, self-representation, rationality, and decision-making [29]. Specifically, cognitive processes in reading are categorized into two: lower level or bottom-up, and higher level or top-down [30]. Both cognitive processes in reading are susceptible to several factors such as reading purpose [11]; readers' text–belief consistency and inconsistency [31]; reading media, whether digital or nondigital [32]; readers' background knowledge [30]; and, most importantly, text difficulty [33]. In their study involving 400 applicants taking the Iranian National University Entrance Examination (INUEE), Baghaei and Ravand [33] assumed several cognitive processes in reading comprehension: reading for details, inference, the main idea, syntax, and vocabulary. Among INUEE items that required cognitive processes, inferencing processing was found as the most challenging task for the applicants, while vocabulary processing was the easiest. Inferencing processing usually intensifies in a test that involves complex text difficulty [30]. Another study on 181 native English-speaking adolescents (9–14.83 years) in the United States also revealed that inference questions are the most challenging reading comprehension questions for students [34]. On the other hand, Norouzi et al. [35] conducted a study on 108 high-school Iranians and found that there was no difference between inferential and textual questions in affecting the students’ reading comprehension scores. These empirical studies showed us evidence that there is a contradictory on which cognitive process in reading that put more burden on the students. Regarding inference questions, it is crucial that students, particularly those entering university-level education, master a reading method to overcome the difficulty of this question type.

Overall, cognitive processes and metacognitive awareness are aspects that a reading comprehension test must elicit for the long term [36,37]. Testers should develop reading tests that reveal test-takers’ cognitive and metacognitive skills. A reading test assesses the students' ability in various tasks of reading comprehension. Tasks, and each task demands different cognitive skills. For instance, to answer a main-idea question, students use a cognitive process that entails a strategy. This process differs from the one used for answering vocabulary questions or other question types. The students’ ability to apply a certain reading strategy for a certain reading problem should also be revealed by a reading comprehension test. This skill is crucial for them as it makes them better readers, especially of academic texts. As a consequence of mastering reading comprehension strategies, the students become more skilled readers and able to regulate themselves in using appropriate strategy for a certain reading comprehension question. This ability is called metacognitive reading skill.

### 2.3 Text variable: textual complexity

In a reading test, textual complexity is linked to the language characteristics of the source test (passage) that should be consistent with those of the target test (task) [18,38]. Barrot [16] divided textual complexity into lexical and syntactic, with the first contributing more to reading comprehension than the latter. Likewise, Reed and Kershaw-Herrera [17] formulated textual complexity with word frequency and sentence length as indicators. Rarely occurring words and long sentences are challenging to process.

As a textual complexity dimension, text readability has been one of the text characteristics that inform the legibility level of a reading text [39,40]. Several literatures have addressed issues regarding textual complexity. First, many studies have compared the readability of passages in school textbooks to that of the national English examination [41,42]. However, attempts to align the complexity of texts taught in schools with those used in national examinations have frequently resulted in disparities. For instance, a study of 155 prescribed school textbooks in Thailand found that the passages in the textbooks were easier to understand than those on the actual national examination. This indicated that discrepancies in text difficulty are a driving factor for Thai students' low reading comprehension in the national examination, while other reader and text characteristics might also play a role. Second, a textual complexity analysis was also done to reading texts of annually held high-stake reading exam (National Examination of English Test) in China for a twenty-five-year span [43]. The study reported that the tests' textual complexity has increased gradually throughout the years, especially in terms of lexical complexity. The increase of the textual complexity involved low word occurrence and more academic vocabularies. The textual complexity gradual increase of the same reading test over the years implied a better reading ability of the current generation. Third, a study conducted at a U.S. community college on ESL and native students' entrance to higher education suggested that testers must tolerate text difficulty level for ESL students [44]. This means that textual complexity of reading texts for native readers is relatively distinct from that of foreign language readers. The above three issues gave us more insight that textual complexity in a reading comprehension test is deemed necessary to manage as a consideration for testing students’ reading comprehension. These empirical studies also indicated that it is crucial to study to what extent degree reading texts in a reading comprehension test affect the level of challenge of the reading test itself. The above studies presented us with the issue of investigating how reading cognitive tasks influence test difficulty and how we adapt the reading levels of native readers to those of foreign readers.

The more diverse the lexis, the less readable the text [3,17]. Poor management of text difficulty level risks the construction of a reading comprehension test consisting of texts mismatched to the test-taker's English proficiency level. Most text readability studies have focused only on the interaction between textual complexity and readers' reading comprehension as individuals [40,[44], [45], [46], [47]] and between the textual complexity of school textbooks and texts of the national examination test [41,42,48]. The gap that this study attempts to fill pertains to the lack of attention to the interaction between the textual complexity of passages in a reading comprehension test and item difficulty in the test. Put simply, the extent to which textual diversity contributes to reading test difficulty has been vague in readability studies. Hence, the current research seeks to adjust textual complexity in a reading comprehension test based on test-takers’ characteristics as undergraduate EFL readers [49]. After this adjustment, the present study examined the interaction between text difficulty and test difficulty. For the long term, this study hypothesizes that texts with higher complexity always undergo complex cognitive processes indicated by the question items in a reading comprehension test.

### 2.4 Objectives of the present study

This study considered reader and text variables [28]: cognitive processes and metacognitive awareness for the former and textual complexity and test difficulty measures for the latter. Some studies used Rasch model analysis to measure the cutoff balance between students’ responses to item difficulty based on item response theory (IRT) [2,3]. The present study investigated the interaction between the textual complexity of the passages in the reading comprehension test and the overall item difficulty levels. Castello [38] argued that reader variables, reading text, and tasks are interdependent, while Barrot [16], Oakland and Lane [18], and Reed and Kershaw-Herrera [17] considered text readability in preparing their respective reading comprehension tests. However, the studies that investigate the interaction between text complexity and reading tasks following the text have been relatively rare. That was the reason why we conducted this study. In addition, based on the three-part model and heuristic comprehension model, we tried to provide evidence that textual complexity, reader, and task interaction could be used as a consideration in reading comprehension test development. To bridge the gap, we formulated the following research questions.

A. Do the items of the reading comprehension test fit the Rasch model?
B. Does item difficulty confirm the passages' textual complexity?
C. What cognitive process contributes the most to the overall item difficulty?

## 3 Method

### 3.1 Design

The study was conducted in line with University of Szeged recommendations and was explicitly authorized by the Institutional Review Board (IRB) of the Doctoral School of Education, University of Szeged. The informed consent was also obtained from all the participants stating that they agreed to participate in the study.

The reading comprehension test was administered online using Google Forms. The instructors in each university spent a few minutes explaining the test online before sending the test link to the students to anticipate any misunderstandings. The questionnaire was anonymous, and no information may be traced to the individual who provided it. The data would also be kept confidential. The test-takers spent 45 min completing the test under their instructors’ supervision. After completing doing the test, the test-takers clicked on the submit button. A response email would be automatically sent to the test-takers respective email address to inform them about their test results.

### 3.2 Participants

The study recruited 1000 undergraduate students from 13 state and private universities across 9 Indonesian provinces (768 females (76.8%) and 232 males (23.2%). We chose Indonesian undergraduate students from various disciplines, as they are generally EFL students who use English exclusively in formal academic settings. Some were English majors with minimal exposure to the language outside their academic context. In Indonesia, undergraduate students usually enroll in English programs throughout their first two years of study, with such courses focusing on English for specific purposes according to their degrees. However, this study classified them as same-level EFL students, as they lacked active utterances outside the classroom. Based on Leather and Uden's [49] description of Indonesian students' characteristics as EFL learners with around 1220 headwords and 900 h of teaching, we classified the participants as undergraduate EFL learners.

The participants’ self-reported survey responses indicated various English proficiency levels according to the Common European Framework of Reference for Languages (CEFR): A1 (22.1%), A2 (29.2%), B1 (14%), B2 (26.8%), C1 (6.5%), and C2 (1.4%). Meanwhile, their responses to the question on attitudes toward English placed them in one of three categories: not liking English (2.8%), possibly liking English (30.2%), and liking English (67%). The participants represented different university majors.

The participants' linguistic differences were deemed insignificant since most of them already received formal English language instruction throughout their previous education. Most of them understood the subject + verb concept without further improvements in other learning areas. Additionally, they seemed to lack metacognition and cognitive awareness to help them self-regulate their language learning and reading comprehension. Bessy and Knouse [50] asserted that metalinguistic awareness enables students to consider how they acquire language and how a particular notion functions in language learning. In the current study's respondents, metacognitive and metalinguistic awareness seemed lacking; however, as they are mature students, we assumed they understood the world concept. Their only problem was that, to some degree, their lexical abilities were far behind those of L1 students, especially English headword mastery. Since our participants were mostly at the A1 and A2 levels, the reading test adaptation considered the participants' English proficiency levels to determine which text difficulty levels were appropriate for them.

### 3.3 Instruments

To decide which multiple-choice test to adopt in the study, we based our decision on reader and text variables [28]. As discussed in the participants’ description, we observed that most of them belonged to one category of readers—EFL learners—and most of them were at the A1 and A2 levels according to the CEFR. In addition, they only have limited English headword mastery; their numbers were insufficient to help them reach a 95% understanding of an authentic English text. That is, to achieve a minimum of 95% understanding of an authentic English text, an EFL reader must encounter 1 unknown word in every 20 words; a target of 98% understanding would require 1 unfamiliar word in every 50 [49].

For its instrument, this study adapted a multiple-choice reading comprehension test consisting of 7 short passages and 38 items with 4 answer options. This was based on Nation's [51] assertion that for learners to comprehend an authentic text, they must master at least 8000–9000 headwords. Meanwhile, Leather and Uden [49] described Indonesian undergraduate students as generally having an estimated 1220 headwords and 900 h of teaching. Therefore, we adapted the instrument by combining three-level reading comprehension worksheets for L1 readers taken from readtheory. org: grades 4, 5, and 6. The reading texts of these three grades were assumed to be within the participants' ability levels and could compensate for their lack of linguistic knowledge. An expert in the reading comprehension test evaluated the instrument's content validity by determining whether it covered all cognitive processes required to be demonstrated by the test, such as locating the main idea, answering vocabulary questions, answering inference questions, and locating specific information. Overall, the reading test appeared adequate for assessing the pupils' reading comprehension.

Meanwhile, before implementing the instrument, this study examined the textual complexity of the passages, as it was the most critical factor to assess [48,53]. The text features were broadly classified into two: passage difficulty and item difficulty. When taking a reading comprehension test, a test-taker engages in the cognitive processes necessary to comprehend and resolve the issues posed by the test questions. Cognitive processes take place during passage reading and decision-making associated with problem-solving [54,55].

With regard to the passage difficulty of the reading comprehension test, the seven passages had word counts ranging from 118 to 416 (mean = 265.8, SD = 87.3). We handled their difficulty level through a preliminary analysis of their textual complexities based on several aspects such as genres, text topic and content, and lexical diversity, such as word count, sentence count, sentence length, type–token ratio, syllable, Flesch–Kincaid grade level, Flesch reading ease, and L2 readability [53,56]. Table 1 shows the numeric properties of these aspects. Since the seven texts in our reading comprehension test belonged to levels A1 (1 text), A2 (5), and C2 (1), we were certain that the reading test would match the ability of most of our respondents.

**Table 1:** Lexical diversities of the seven passages in the reading comprehension instrument.

Genre	Text Topic and Content	Word Count	Sen-tence Count	Average Sentence Length	Type–Token Ratio	Words with More than Two Syllables	Flesch–Kincaid Grade Level	Flesch Reading Ease	L2 Reada-bility	CEFR
Narrative	Sport	418	65	6.15 words	0.45	13	2.0	91.6	32.9	A1
Descriptive	Animal	119	12	10.17 words	0.58	6	4.43	81.40	20.3	A2
Narrative	Academic Life	322	25	12.92 words	0.57	64	9.94	46.78	14.9	C2
Narrative	Spending Money	295	29	10.38 words	0.52	14	4.37	82.19	16.3	A2
Argumen-tative	Vacation	223	20	11.25 words	0.48	20	5.21	77.73	24.9	A2
Narrative	Birthday	289	27	10.85 words	0.45	23	4.35	83.21	20.2	A2
Narrative	Broke	212	22	9.68 words	0.55	23	4.14	82.62	31.1	A2

The texts' lexical diversities were calculated using textinspector.com, cohmetrix.com, and lextutor.ca.

For the text genre, the more narrative a text, the easier it is to comprehend [53]. The seven passages comprised five narrative texts, one descriptive text, and one argumentative text. However, based on the sequence in Table 1, the third text was the most complicated of all the passages, which was not surprising since the textual diversity of its content indicated more difficulty levels than the other texts. Meanwhile, for the non-narrative texts, text 5 and text 2 were in the second and third positions in Flesch–Kincaid’s grade level, respectively. The genres of both texts that were not narrative could contribute to their difficulty. Therefore, we assumed that text genres and lexical diversity properties separately account for text difficulty.

Text topic and content could be an attribute of difficulty level. Alderson [28] argued that a nonspecialist social science test would be easier to understand by regular people of equivalent educational attainment. All the passages in our instruments discussed general topics and contents and were assumed to tap into the participants’ schemata to easily comprehend the text.

Concerning the students’ potential difficulty in solving the problem posed by each instrument item, the kinds of cognitive processes we expected the participants to implement while taking the test could be a significant source of test difficulty. The cognitive processes in our adapted reading comprehension test were finding the main idea, answering vocabulary questions, using detailed information in the text, and inferring [33].

### 3.4 Data analysis

While the study focused on confirming whether the passages' difficulty level in reading comprehension was consistent with test difficulty, we performed Rasch model analysis to assess the trade-off of test-takers’ ability and test item difficulty to reveal important information regarding the instrument. Such information was important for more improvements in future research, especially as textual complexity was used in this study as a basis of the instrument. Item difficulty estimates in the Rasch model pertain to the test-taker's response to the items by considering the match between test-taker ability and item difficulty level [57].

One-way ANOVA was performed to analyze two aspects. First was the possible differences in the test-takers’ reading comprehension scores on the passages' textual complexity. We categorized such textual complexity based on the lexical diversity data shown in Table 1. Accordingly, text focusing on sports was placed under the easy category, while those focusing on animals, spending money, vacations, birthdays, and being broke belonged to the medium category. Only one text, academic life, was considered difficult. The second aspect referred to the test-takers’ differences in terms of their reading comprehension performance attributes: finding the main idea, answering vocabulary questions, using detailed information in the text, and inferring [33].

## 4 Results

The items in the instrument had a Cronbach's alpha of .92, indicating high reliability. The dichotomous Rasch model analysis was performed using the SnowIRT package in Jamovi [58]. Our first attempt at model fit resulted in the elimination of six items, which had fewer contributions to item fitness. We initially tested the 38 items of the reading comprehension test. We used a mean-square fit value of between 0.5 and 1.5 [59], as the test belonged to a low-stake test category. Six items, that is, items 4 (MnSq = 1.51), 12 (MnSq = 1.72), 13 (MnSq = 1.74), 14 (MnSq = 2.31), 15 (MnSq = 3.22), and 22 (MnSq = 1.77), were found to misfit the Rasch model for having mean-square results >1.5. The remaining 32 items were then retested to find the best fitness within the accepted range. Each item's difficulty level was an important factor that affected the proportion of items answered correctly by the test-takers. Based on IRT, the probability of a correct response to a question results from a mathematical function between a test-taker's ability and item parameters [60]. Because the mean of the difficulty level was 0, a test-taker with 0 means of ability level would have a 50/50 chance to answer the item correctly. Based on the analysis, only items 14 (estimate = 0.27, SE = 0.07), 15 (estimate = 0.89, SE = 0.07), and 20 (estimate = 0.006, SE = 0.07) had measures above 0, while the others had measures below 0. This means that the majority of the items in our instrument were easy for the test-takers. The mean-square fit statistics were close to 1, which was within the acceptable range of > 0.5 to <1.5.

At least two essential insights were drawn from the Wright map in Fig. 1. First, regarding one aspect in the item parameter, the difficulty estimates of the items mostly ranged below the 0 logit, indicating that most of the items were easy. According to the group, item 15 (estimate = 0.89, SE = 0.07) at the upper end was the most difficult item, while item 3 (estimate = ˗ 3.14, SE = 0.12) was the easiest. Since several items were found along with students of lower ability, many were below the overall difficulty. Second, redundant areas were found at five different lines consisting of more than two items per line, indicating that the items may measure the same difficulty level.

Moreover, our instrument underwent the Andersen likelihood-ratio test using the eRM package in R, resulting in an LR value of 359.89, a chi-square *df* of 31, and a p-value of 0. This indicated a violation of the items’ local independence as well as a one-dimensionality risk in the instrument.

Before performing the ANOVA analysis on the influence of the passages’ Flesch–Kincaid grade levels on test difficulty level, we assumed three grade levels of the passages based on the Flesch–Kincaid grade level analysis and readability formula as well as our assumption on test-taker ability. A narrative text about sports (Flesch–Kincaid grade level = 2.0, mean = ˗1.95, SD = 1.04) was the easiest or most appropriate for A1-level readers, while a narrative text about academic life (Flesch–Kincaid grade level = 9.94, mean = −0.92, SD = 0.64) was considered the most challenging or appropriate for C2-level readers. The remaining five passages (Flesch–Kincaid grade levels = 4.14–5.21, Mean = −0.91, SD = 0.47) matched the ability of A2-level readers.

In addition, a one-way ANOVA (Table 2) showed a significant difference in reading comprehension scores based on the six subsample categories, *df* = 994 (p < .05), but because equal variance was not assumed between groups and *df* = 5 (p < .05), a post hoc test using Dunnett's T3 was performed. The pairwise comparison test showed that C1 and C2 test-takers differed from the rest in terms of reading comprehension scores. This analysis provides evidence that the adapted reading comprehension test differentiates test-takers according to their proficiency levels.

**Table 2:** Reading comprehension differences based on subsamples.

Subsamples Ability Levels	Measures		*F* (994)
	M	SD	
A1	20.71	8.04	7.21*
A2	21.40	8.04	
B1	21.20	7.92	
B2	20.67	7.77	
C1	24.78	7.46	
C2	30.79	1.76	

*P < .05.

In another one-way ANOVA (Table 3), the seven passages in the reading comprehension test belonging to different grade levels significantly contributed to the test difficulty measure. Although the majority of the items were considered easy based on the test-takers’ ability levels, a significant difference was observed in the test difficulty measures based on the three text difficulty levels, df = 29 (p < .05). Equivalent homogeneity of variances between groups, *df* = 2 (p > .05), involved a post hoc test using Tukey's b, which meant that no text grade level differed from the others. In future research, a more balanced number of text levels might generate a more prominent significant effect on reading comprehension difficulty.

**Table 3:** Item measure differences based on Flesch–Kincaid grade level.

Flesch–Kincaid Grade Level	Measures		*F* (29)	Ptukey
	M	SD		
A1	˗1.95	1.04	3.96*	˗1.95
A2	˗.92	.64		˗.92
C2	˗.91	.47		˗.91

*P < .05.

The final one-way ANOVA analysis (Table 4) showed insignificant differences in test difficulty levels based on reading comprehension subskills, *df* = 3 (p > .05). However, reading for inference (mean = −0.68, SD = 0.72) ranked the highest of all subskills, while vocabulary (mean = ˗1.29, SD = 1.00) ranked the lowest. Reading for the main idea (mean = ˗1.19, SD = 0.47) and reading for details (mean = ˗1.23, SD = 0.72) were at the same difficulty level. More balanced subskills used in future studies might result in reading comprehension test differences.

**Table 4:** Item measure differences based on reading comprehension subskills.

Reading Comprehension Subskills	Measures		*F* (3)
	M	SD	
Main Idea	˗1.19	0.47	1.344*
Vocabulary	˗1.29	1.00	
Details	˗1.23	0.72	
Inference	˗0.68	0.72	

*P > .05.

## 5 Discussion

The purpose of this study was to investigate the interaction between the text complexity and degree of difficulty of the tasks, following each text in an adapted reading comprehension test. This study had an adequate sample size and assumed the one-dimensionality of the reading test, which confirmed two requirements to perform a Rasch model analysis [61]. The Alderson likelihood-ratio test using the eRM package in R found that *p* = .000 Baghaei and Kubinger [2] stated that if *p* < .5, there is a violation of instrument one-dimensionality in that local item dependence (LID) is observed among items. Tuerlinckx and De Boeck [62] stated that the items might interact because of several questions regarding a particular reading comprehension passage.

### 5.1 Do the items of the reading comprehension test fit the Rasch model?

Initially, we eliminated the misfit items based on their score residual, which indicated excessively predictable items and noticeable off-variable noise, as discussed in Holster et al. [3]. However, most of the remaining 32 fit items were overly predictable, which led us to overestimate the instrument's measurement capability. We understand that items with negative values are deemed too predictable; however, predictability is a mere label, and it is up to the researcher to determine the appropriate course of action [63]. Nevertheless, because of its high reliability, the reading comprehension instrument can consistently measure what it is supposed to measure. However, future instrument development and adaptation must consider the primary issue from two critical aspects of our findings—low item estimates and redundancy concerns.

**Fig. 3:**
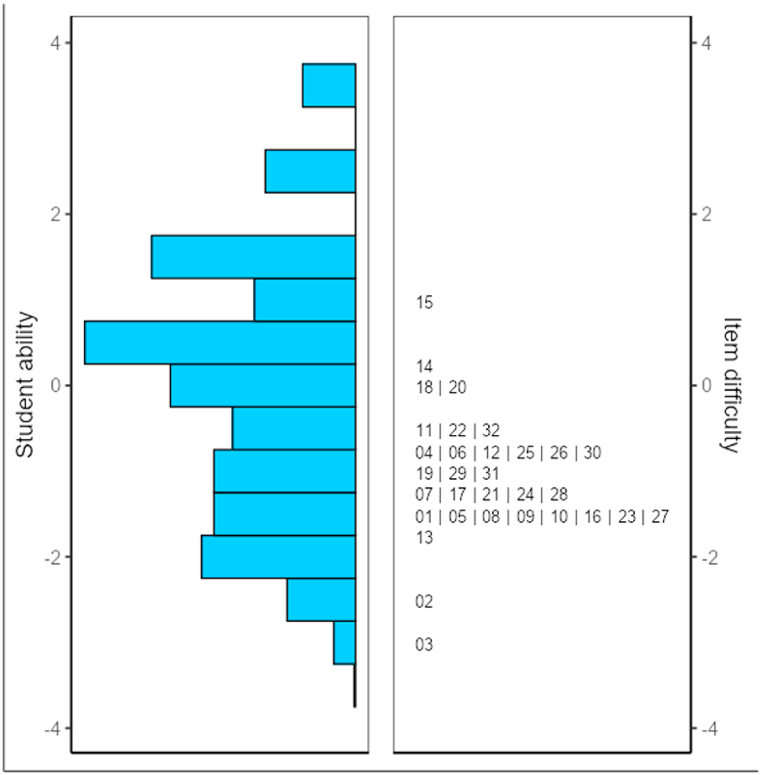
Item-by-person mapping in the Wright map.

The low difficulty estimates for the items relative to students' ability may be due to a mismatch between the instrument's ability to assess test-takers’ reading ability and the test-takers’ actual ability. Before developing the instrument, the test-takers’ average ability must be evaluated more thoroughly. This would entail item preparation, that is, enabling a tester to match the test difficulty level to test-takers’ ability, which we followed when we were developing our reading comprehension instrument. Considering Leather and Uden's [49] estimate of Indonesian undergraduate students' average mastery of 1220 headwords with 900 h of English class in their previous education and the heterogeneity of our undergraduate student sample from various fields of study, we lowered our passages standard to native readers' reading levels of grades 4, 5, and 6. We assumed that the majority of our sample participants experienced difficulty with their linguistic knowledge and chose texts appropriate for their ability level. However, as indicated by our Wright map (see Fig. 3), the test results did not seem to confirm our prediction regarding test-takers’ ability. Most items were easy for our sample. Test-takers’ breadth of vocabulary, assumed at a 1220-headword mastery, was superior to the passages in our instrument, whose unique words ranged from 69 to 184. This necessitates a readjustment of the passages' textual complexity through a selection of texts with more unique/type words. Crossley et al. [64] discussed text adaptation for ESL learners using familiar genres, such as narrative text, as well as textual complexity adaptation, especially lexical and syntactical substitution in the adapted text. Furthermore, Jin and Lu [65] and Jin et al. [66] suggested that an increase in difficulty level during text adaptation is mostly associated with lexical and syntactical changes in the text. The reality, however, is that no clear guidelines and evidence have been offered regarding text adaptation [67]. The fact that the texts were too easy for our test-takers also indicates that we cannot solely base our estimate of their proficiency levels on their self-reported responses to our questionnaire. Instead, a more clinical approach should be adopted to examine test-takers’ proficiency levels in depth, especially in smaller environments such as classrooms.

The redundancy issues in our instruments may have been due to overly predictable items that assessed the same ability [68]. To avoid such redundancy, Nolte et al. [69] argued that item removal could be implemented. Overall, item redundancy could be indirectly caused by the pandemic situation, as we could only collect data online by distributing our reading comprehension test via Google Forms.

Most of the study participants completed the reading comprehension test independently at home and could do so with external help. Another possible explanation is that our respondents were reluctant to take the test and thus recklessly answered the instrument's questions. Because of the pandemic, our psychometric analysis may be skewed, as the observed results differed significantly from the expected ones given the degree of item difficulty. A set of redundant items, that is, numbers 4, 6, 12, 25, and 26, measured the same subskill: reading for details. Only item 30 measured reading for inference. Meanwhile, items within the same line, that is, numbers 1, 5, 8, 9, 10, 16, 23, and 27, measured different subskills equally: reading for the main idea, vocabulary, and reading for details. The adapted test's usability and practicality may be achieved to a certain degree because of its ability to differentiate test-takers according to their proficiency levels, as shown in Table 3.

The items in the instrument fit the Rasch model since each observed item approximated the expected trend. However, future studies must examine the two critical issues mentioned above for better instrument development. The findings confirm our theory that a tester must focus on textual complexity when constructing a reading comprehension test [1,20,21,28,48,49,53]. In particular, the Rasch analysis that we performed represented the consideration of reader and the task as in the three-part model by RAND Reading Study Group. Likewise, the textual complexity management that we did on the texts in the reading comprehension test represented the quantitative dimension of the text complexity as in Ref. [19]. With the items fit the Rasch model, we believe that the three-part model worked well in our reading comprehension test adaptation. Hence, the use of textual complexity analysis and management in developing a reading comprehension test can be adopted by EFL reading teachers.

### 5.2 Does item difficulty confirm the passages’ textual complexity?

After performing Rasch analysis, we found that most of the items in the reading comprehension test were easy for the participants. However, when we conducted a one-way ANOVA on item differences based on textual complexity, we observed differences in reading comprehension difficulty based on the text's difficulty levels. The reading questions following the more complex texts tended to be more challenging than those of the easier texts. This result confirms our hypothesis that the more complex the passage in a reading comprehension test, the more complex the questions or tasks that follow. This finding strengthens Castello's [38], and Oakland and Lane's [18] argument that reader variables and reading text and tasks are all inextricably linked.

Our findings further support the notion that in any reading comprehension test, textual complexity is a significant factor that influences readers' comprehension of reading texts [16–18]. The most difficult text used in the test, according to the student responses, was a descriptive text with an academic topic. It was a C2 level text with 12.92-word length, and the type–token ratio was 0.57. The text had the least Flesch reading ease and the lowest L2 text readability indices relative to the other texts. The text complexity resulting from the higher type–token ratio influenced the students’ comprehension as well as the solution needed to solve the tasks. Moreover, the findings about the linearity between text complexity and task difficulty gave added support to our main theory by Amendum et al. [1] that text difficulty is an accumulation of the interaction between the text complexity, reader, and task difficulty. Based on the results, we assumed that the significant difference between test items would be more apparent if the texts were more complex and the number of subskill tasks following each passage were balanced.

### 5.3 What cognitive process contributes the most to overall item difficulty?

It is critical to conduct research on reading comprehension subskills before engaging in the practice of teaching reading comprehension through finer details. Reading scholars have proposed numerous subskills of reading comprehension, such as vocabulary processing [70]; textual complexity and cognitive processes for reading [71]; and reading for main idea, inference, vocabulary, details, and syntax [33]. The current study used ANOVA to test several subskills and found that these subskills did not significantly affect the difficulty of the reading comprehension test. However, a closer examination of the results revealed that reading for inference was more difficult than the other subskills, while answering vocabulary questions remained the most manageable problem for test-takers to solve, confirming Baghaei and Ravand's [33] cognitive processing model in a reading comprehension test. This result was significant because it supported the theory that students have more difficulty with a top-down reading process like inferencing in comparison to a bottom-up reading process [30]. Also, this study supported Spencer's et al. [34] findings that even among native adolescent readers, inference questions in reading demanded deeper cognitive process. At the same time, this study contradicted the similar study in the Iranian context which found that there was no difference between the effect of inferential questions and textual questions on students' reading comprehension scores [35]. However, no matter which cognitive processes in reading matter the most for the students, preparing them with appropriate cognitive strategies in reading is recommended. Therefore, it is important to work more on a strategy for inferencing to help students shape their metacognitive strategies as they become more adept with cognitive strategies in reading [36,37].

## 6 Implication

The results of our study have various implications for future research into the consideration of textual complexity in the construction of reading comprehension tests [1,20]. While identifying the students' individual differences in reading comprehension during the initial diagnostic analysis is crucial for matching the reading test's difficulty level to each student's reading ability, it is also useful to know how much the reading text's complexity affects the difficulty of the reading questions. Different reading tasks give different difficulty dimensions in reading questions. This study provides evidence that text complexity contributes to the task difficulty level in a reading comprehension test.

This study also gives implication for practical aspect of testing the students’ reading comprehension. By determining which cognitive function contributed the most to the difficulty level of the reading comprehension test, teachers will be able to assist students with specialized problem-solving strategies [33]. Students find inference questions to be the most difficult on nearly every reading comprehension test. Therefore, offering pupils a method for answering inference questions is a prudent action for teachers to do. In addition, by mastering all reading methods, students can mold their metacognitive strategies to become better readers.

## 7 Conclusions

This study proposed two hypotheses based on Alderson's [28] theory regarding the relation between reader and text variables in reading comprehension assessment. First, the higher the textual complexity in a reading comprehension test, the more challenging the tasks that follow. Second, test-takers’ cognitive and metacognitive skills contribute to reading comprehension difficulty. Based on the current findings, we discuss several limitations and offer possible solutions for future research.

We based the current study's limitations on three general problems we diagnosed from the data analysis. The first was the test's insufficient difficulty level. Initially, we used data on the test-takers’ proficiency levels and headword mastery generalizability by Leather and Uden [49] to adapt a reading comprehension test. Using the textual complexity approach, which covered genres, text topic and content, word count, average sentence length, type–token ratio, and words with more than two syllables, we assumed that student worksheets for grades 4, 5, and 6 from readtheory.org were appropriate for our sample. We defined each passage's difficulty as represented by Flesch–Kincaid grade level, with one difficult text, one easy text, and five medium texts. However, results of the Rasch analysis revealed that most items were easy relative to the test-takers’ ability. The solution here would then be to increase the test's difficulty level to a certain degree while focusing on reader and text variables.

The second issue was LID. After conducting Rasch analysis on the instrument data, we found that most items depended on the other items. This could be due to the imbalance of items in each subskill; hence, answering a certain item depended on answering other items. For instance, there were too many items in reading for inference and reading for details. Another possible cause was the pandemic situation, which may have distorted the data collection process in that the university instructors found it nearly impossible to monitor the test-takers (students) as they were completing the reading comprehension test. As a result, some students might have been reluctant to take the test and may have done so hastily. The possible solution here would be more effort toward balancing the number of items in every processing skill applied in a reading comprehension test, as well as a more collaborative approach adopted with instructors as helpers in conducting research.

The last problem was item redundancy. In the analysis (see Fig. 1), five lines of items probably measured the same level of test-takers’ ability. Item redundancy could have been due to the imbalance in the number of items and the overly easy tasks assigned to test-takers. The possible solution to this issue is to increase the test difficulty level to a certain degree and strike a balance in the item count for each applied subskill.

Finally, we suggest that future research that attempts to match reader, text complexity, and task should be rigorous in evaluating each student's capacity. Therefore, sufficient information for the instructor to determine the text's level of difficulty will be included in the reading test. Future research should further broaden the reader-text-task match study in secondary schools, particularly in terms of comparing the text complexity of textbooks and examinations. This study shows that undergraduate EFL instructors may develop or adapt a reading comprehension test with a level of difficulty that matches the students' ability. Reader and text variables are two important aspects to consider when managing the textual complexity of passages in a reading comprehension test. Although considerable effort is required to construct the most appropriate test for students' proficiency levels, managing textual complexity does help instructors predict possible instrument problems and identify which cognitive processes and strategies must be emphasized in reading comprehension training. The textual complexity practice example in this study, as well as the identified limitations and solutions, are significant for EFL reading comprehension and its related issues and development in Indonesia and other similar contexts.

## Declaration

### Author contribution statement

Helta Anggia: Conceived and designed the experiments; Performed the experiments; Analyzed and interpreted the data; Contributed reagents, materials, analysis tools or data; Wrote the paper.

Anita Habók: Conceived and designed the experiments; Analyzed and interpreted the data; Contributed reagents, materials, analysis tools or data.

### Funding statement

This work was supported by the University of Szeged Open Access Fund (grant number: 5843) and the Research Programme for Public Education Development, Hungarian Academy of Sciences (grant KOZOKT2021-16).

### Data availability statement

The data that has been used is confidential.

### Declaration of interest's statement

The authors declare no competing interests.

## References

[1] Amendum S.J., Conradi K., Hiebert E. (2018). Does text complexity matter in the elementary grades? A research synthesis of text difficulty and elementary students' reading fluency and comprehension. Educ. Psychol. Rev..

[2] Baghaei P., Kubinger K.D. (2015). Linear logistic test modeling with R. Practical Assess. Res. Eval..

[3] Holster T., Lake J., Pellowe W. (2017). Measuring and predicting graded reader difficulty. Read. Foreign Lang..

[4] Baba Öztürk M., Aydogmus M. (2021). Relational assessment of metacognitive reading strategies and reading motivation. Int. J. Prog. Educ..

[5] Habók A., Magyar A. (2018).

[6] Oo T.Z., Habók A. (2020). The development of a reflective teaching model for reading comprehension in English language teaching. Int. Electron. J. Environ. Educ..

[7] Oo T.Z., Habók A. (2022). Reflection-based questioning: aspects affecting Myanmar students' reading comprehension. Heliyon.

[8] Wang L., Wang J., Liu D., Lin D. (2021). The role of metalinguistic awareness and character properties in early Chinese reading. J. Exp. Child Psychol..

[9] Kaya S., Eryilmaz N., Yuksel D. (2022). The effects of motivational and metacognitive variables on immigrant and non-immigrant students' reading achievement. Int. J. Educ. Res..

[10] Sheorey R., Mokhtari K. (2001). Differences in the metacognitive awareness of reading strategies among native and non-native readers. System.

[11] Mesgarshahr A. (2019). Understanding L2 reading cognitive processes : the case of the L2 reader. ’ s Goal’.

[12] Chen S., Du H., Wang S., Yang L. (2022). Understanding EFL reading anxiety in relation to learning motivation, attitudes and strategies for Chinese and Spanish undergraduates. System.

[13] Alderson J.C., Nieminen L., Huhta A. (2016). Characteristics of weak and strong readers in a foreign language. Mod. Lang. J..

[14] Zou X.L., Ou L. (2020). EFL reading test on mobile versus on paper: a study from metacognitive strategy use to test-media impacts. Educ. Assess. Eval. Account..

[15] Gkikas D.C., Tzafilkou K., Theodoridis P.K., Garmpis A., Gkikas M.C. (2022). How do text characteristics impact user engagement in social media posts: modeling content readability, length, and hashtags number in Facebook. Int. J. Inf. Manag. Data Insights.

[16] Barrot J.S. (2013). Revisiting the role of linguistic complexity in ESL reading comprehension. 3L Lang. Linguist. Lit..

[17] Reed D.K., Kershaw-Herrera S. (2016). An examination of text complexity as characterized by readability and cohesion. J. Exp. Educ..

[18] Oakland T., Lane H.B. (2004). Language, reading, and readability formulas: implications for developing and adapting tests. Int. J. Test..

[19] Shanahan T. (2013). https://www.generationready.com/wp-content/uploads/2021/04/Beginners-Guide-to-Text-Complexity.pdf.

[20] Mesmer H.A., Cunningham J.W., Hiebert E.H. (2012). Toward a theoretical model of text complexity for the early grades: learning from the past, anticipating the future. Read. Res. Q..

[21] RAND Reading Study Group (2002).

[22] Fathi J., Shirazizadeh M. (2020). The effects of a second language reading strategy instruction on iranian EFL learners' reading comprehension and reading anxiety. Lang. Relat. Res..

[23] Ghaith G., El-Sanyoura H. (2019). Reading comprehension: the mediating role of metacognitive strategies. Read. Foreign Lang..

[24] Khonamri F., Kojidi E.M. (2011). Metacognitive awareness and comprehension monitoring in reading ability of Iranian EFL learners. Profile.

[25] Bećirović S., Brdarević-Čeljo A., Dubravac V. (2018). The effect of nationality, gender, and GPA on the use of reading strategies among EFL university students. Sage Open.

[26] Míguez-Álvarez C., Cuevas-Alonso M., Cruz M. (2021). The relationship between metacomprehension and reading comprehension in Spanish as a second language. Psicol. Educ..

[27] Par L. (2020). The relationship between reading strategies and reading achievement of the EFL students. Int. J. Instr..

[28] Alderson J.C. (2000). http://ezproxy.york.ac.uk/login.

[29] Newen A. (2017). What are cognitive processes? An example-based approach. Synthese.

[30] Kendeou P., Muis K.R., Fulton S. (2011).

[31] Maier M.A.B., Johanna, Richter Tobias (2018). Cognitive processes underlying the text‐belief consistency effect: an eye‐movement study. Appl. Cognit. Psychol..

[32] Jian Y.C. (2022). Reading in print versus digital media uses different cognitive strategies : evidence from eye movements during science - text reading. Read. Writ..

[33] Baghaei P., Ravand H. (2015). A cognitive processing model of reading comprehension in English as a foreign language using the linear logistic test model. Learn. Indiv Differ.

[34] Spencer M., Gilmour A.F., Miller A.C., Emerson A.M., Saha N.M., Cutting L.E. (2019). Understanding the influence of text complexity and question type on reading outcomes. Read. Writ..

[35] Norouzi F., Haghverdii H.R., Shafiee S. (2015).

[36] Xu Y., Wong R., He S., Veldre A., Andrews S. (2020). Is it smart to read on your phone? The impact of reading format and culture on the continued influence of misinformation. Mem. Cognit..

[37] Ozturk N. (2019).

[38] Castello E. (2008).

[39] Dirgantari H. (2020). Suci alfi, susantiningdyah, ‘students’ reading comprehension skill: the roles of text readability and question difficulty. Int. J. Soc. Sci..

[40] Vajjala S., Lučić I. (2019). ACL 2019 - Innov. Use NLP Build. Educ. Appl. BEA 2019 - Proc. 14th Work..

[41] Srisunakrua T. (2019). Thanaporn, chumworatayee, ‘readability of reading passages in English textbooks and the Thai national education English test: a comparative study. Arab World Engl. J..

[42] Tunde-Awe B.M., Ogunyemi K.O., Olajide S.B. (2021). English textbook's readability and junior secondary school students' performance in reading comprehension. African J. Appl. Res..

[43] Yu X. (2021). Text complexity of reading comprehension passages in the national matriculation English test in China: the development from 1996 to 2020. Int. J. Lang. Test..

[44] Taylor Z. (2020). College admissions for L2 students: comparing L1 and L2 readability of admissions materials for U.S. Higher education. J. Coll. Access.

[45] Dirgantari A.S., Susantiningdyah H. (2020). Students' reading comprehension skill: the roles of text readability and question difficulty. Int. J. Soc. Sci..

[46] Hakim A.A., Setyaningsih E., Cahyaningrum D. (2021). Examining the readability level of reading texts in English textbook for Indonesian senior high school. J. English Lang. Stud..

[47] Handayani R., Furaidah F., Ivone F.M. (2021). The readability level of reading texts in erlangga straight point series: English for eleventh grade students. J. Pendidik. Teor. Penelitian, dan Pengemb..

[48] Cárcamo Morales B. (2020). Readability and types of questions in Chilean EFL high school textbooks. TESOL J..

[49] Leather S., Uden J. (2021). ‘Extensive Reading’, *Extensive Read.*.

[50] Bessy M., Knouse S. (2020). Metacognition, metalinguistic awareness, and relevance in language learning: a report on an intervention module project. Int. J. Scholarsh. Teach. Learn..

[51] Nation I.S.P. (2006). How large a vocabulary is needed for reading and listening?. Can. Mod. Lang. Rev..

[53] Solnyshkina M.I., Zamaletdinov R.R., Gorodetskaya L.A., Gabitov A.I. (2017). Evaluating text complexity and Flesch-Kincaid grade level. J. Soc. Stud. Educ. Res..

[54] Ali A.M., Razali A.B. (2019). A Review of studies on cognitive and metacognitive reading strategies in teaching reading comprehension for ESL/EFL learners. Engl. Lang. Teach..

[55] Hahnel C., Goldhammer F., Kröhne U., Naumann J. (2018). The role of reading skills in the evaluation of online information gathered from search engine environments. Comput. Hum. Behav..

[56] Zamanian M., Heydari P. (2012). Readability of texts: state of the art. Theor. Pract. Lang. Stud..

[57] McNamara D.S., ’ (2001). Cesare cornoldi and jane oakhill. J. Pragmat..

[58] Project T.J. (2021). http://www.jamovi.org.

[59] Linacre J.M. (1994).

[60] Mair P., Hatzinger R. (2007). Extended rasch modeling: the eRm package for the application of IRT models in R. J. Stat. Software.

[61] Sick J. (2010). Rasch measurement in language education Part 5: assumptions and requirements of Rasch measurement. JALT Test. Eval. SIG Newsletter..

[62] Tuerlinckx F., De Boeck P. (2001). The effect of ignoring item interactions on the estimated discrimination parameters in item response theory. Psychol. Methods.

[63] Boone W.J., Yale M.S., Staver J.R. (2014).

[64] Crossley S.A., Louwerse M.M., McCarthy P.M., McNamara D. (2007). A linguistic analysis of simplified and authentic texts. Mod. Lang. J..

[65] Jin T., Lu X. (2018). A data-driven approach to text adaptation in teaching material preparation: design, implementation, and teacher professional development. Tesol Q..

[66] Jin T., Lu X., Ni J. (2020). Syntactic complexity in adapted teaching materials: differences among grade levels and implications for benchmarking. Mod. Lang. J..

[67] Crossley S.A., Allen D., McNamara D.S. (2012). Text simplification and comprehensible input: a case for an intuitive approach. Lang. Teach. Res..

[68] Byrne B.M. (2005). Factor analytic models: viewing the structure of an assessment instrument from three perspectives. J. Pers. Assess..

[69] Nolte S., Coon C., Hudgens S., Verdam M.G.E. (2019). Psychometric evaluation of the PROMIS® Depression Item Bank: an illustration of classical test theory methods. J. Pat. Repor. Outcom..

[70] Zhao A., Guo Y., Biales C., Olszewski A. (2016). Exploring learner factors in second language (L2) incidental vocabulary acquisition through reading. Read. Foreign Lang..

[71] Nahatame S. (2021). Text readability and processing effort in second language reading: a computational and eye-tracking investigation. Lang. Learn..

