# Supplementary material for: Textual complexity adjustments to the English reading comprehension test for undergraduate EFL students
Source: Heliyon. 2023 Jan 10;9(1):e12891. doi: 10.1016/j.heliyon.2023.e12891 (PMC9868442; doi:10.1016/j.heliyon.2023.e12891)
Supplement: Multimedia component 1 [file mmc1.docx]

Reading Comprehension Test

**Cody Likes to Run**

Cody likes to run. So does his mother, April. Sometimes, Cody and April like to run together. But they like it for different reasons.

Cody is very good at running. He is very fast. He loves competition. On the playground, he participates in races. He almost always wins in the 50-yard dash! One day, a new student came to the school. Everyone thought he looked very fast. But when he raced, Cody beat him. Cody enjoys winning and being good. This is the main reason he likes running. He is so good at it!

Cody likes being good at running. But April likes running for different reasons. April likes the benefits of running. Running helps her stay healthy. It also makes her heart strong. And it helps her feel happy. This is why April likes running. It makes her feel good!

Cody likes to sprint. He likes to run very fast. He likes how exciting it is. He becomes excited when the starter yells, “On your marks, get set, go!” He is good at starting at the right moment.

April enjoys distance running. This means that she does not try to run fast. She tries to run far. Usually, she runs more than a mile. Sometimes, she runs five or six miles! She runs them nice and slow.

One Saturday the school held a race. It was a 3-mile race. Students and parents were both invited to run. Cody really wanted to race his mother. “Please, can we go race?” Cody boasted he would beat his mother! But April just smiled.

So they went to the race. The starter yelled, “On your marks!” Cody was ready. The race began! Cody started out running fast. He ran the first mile. He looked back to see his mother. He was way ahead of her! During the second mile, Cody slowed down. His mother was catching up! Now, she was passing him! “Come on, slow-poke,” she teased. Cody dug in and ran really hard. But he could not keep it up! He was getting a cramp! Soon, April crossed the finish line. She looked back to see Cody. He was coming along slowly. “You can do it!” she shouted. Cody heard her and smiled. He headed for the finish line. He was breathing heavy. Finally, he made it!

“Not too bad for an old lady?” said April.

Cody hugged her. “Hey mom,” Cody said. “Will you teach me to run like you?” April smiled and said, “Of course.”

**1) The main purpose of paragraph 3 is to**

A. talk about how to make your heart strong

B. explain why April likes running

C. tell the reader Cody’s mother’s name

D. show that April and Cody have the same attitude toward running

**2) In paragraph 3, we learn that "April likes the benefits of running." Using this information as a guide, we can understand that benefits**

A. scare you

B. hurt you

C. anger you

D. help you

**3) According to the passage, how long is the school race?**

A. 1 mile

B. 2 miles

C. 3 miles

D. 4 miles

**4) In paragraph 6, why does April most likely smile when Cody boasts that he will beat her?**

A. She knows he is right.

B. She is planning to let him win.

C. She feels proud because Cody has been practicing for this race.

D. She knows she is better at distance running than Cody is.

**5) How does Cody run in the race?**

A. He starts out running very fast, then gets tired and finishes slow.

B. He gets a cramp right away, but runs the last mile very fast.

C. He is careful not to run too fast too soon.

D. He stays near his mother for the first two miles, and then falls behind.

**Camels**

Camels are some of the world’s most interesting creatures. Some camels have one hump on their backs. These are called dromedary camels. Other camels have two humps on their backs. These are called Bactrian camels. Camels are the only animals with humps. Many people believe that camels use their humps to store water, but that is false.

Camels actually use their humps to store fat. A well-fed camel’s hump can weigh up to 80 pounds! Humps allow camels to live up to two weeks without needing to eat. Camels live in the desert, where food and water is scarce. A camel's humps keep it alive and well during times when food and water are far away or unavailable.

- 1. **6) What are camels with two humps on their backs called?**
  2. A. Dromedary camels
  3. B. Bactrian camels
  4. C. Two-hump camels
  5. D. Desert camels
  6. **7) Camel humps are used to store**
  7. A. fat
  8. B. muscle
  9. C. milk
  10. D. water
  11. **8) As used in the passage, the word scarce most nearly means**
  12. A. healthy and delicious
  13. B. frightened
  14. C. very expensive
  15. D. hard to find

**Diligence and Perseverance**

Elizabeth was brooding in her room. She had sought asylum there since spurious gossip about her began circulating at Seagrove Academy last week.

Not that Elizabeth had ever been considered a social butterfly; she had always had a rather reserved personality. Elizabeth preferred to live vicariously through the stories of her more brazen friends: late night partying, fraternizing with boys, childish pranks. Still, she had taken to being more by herself than usual since the allegations surfaced. You see, a student had given headmaster Billings an anonymous "tip" that Elizabeth had cheated on several tests this year. What made this allegation especially damaging was that Elizabeth had recently been nominated to receive the highly coveted Blauvelt Award, a scholarship recognizing "academic integrity and promise."

The accusations were laughable. Elizabeth had long been a stellar student at Seagrove. She lacked a natural intelligence—this was true. However, she compensated for this deficit through diligence and perseverance; she was very thorough in her studies and exhibited an almost relentless determination.

Still, the accusations had given the recommendation committee pause. On Friday Elizabeth had been called to Mr. Billings’s office, where she was questioned about her performance on recent exams. The experience was quite traumatic.

Seagrove was an elite school; most of its students came from privileged backgrounds. But this was not the case for Elizabeth. Her family had little money. She attended Seagrove on a full scholarship. The Blauvelt Award would help Elizabeth's family pay for her college.

So it was with the same diligence that she applied to her studies that Elizabeth planned to expose her accuser. She opened the school directory on her bed and began combing through the names.

Seagrove was such a small and insular community. A mere twenty-one kids would be in her graduating class. Elizabeth knew it was inevitable that the person spreading rumors about her would come to light. It was just a matter of time.

**9) As used in paragraph 1, which is the best synonym for spurious?**

A. hurtful

B. terrible

C. serious

D. untrue

**10) It can be inferred that Elizabeth would have likely been disqualified from receiving the Blauvelt Award if caught cheating because**

A. Mr. Billings said this was the case

B. awards are never given to cheaters

C. the Blauvelt Award is based on academic integrity

D. cheating would have made the award ineligible for college use

**11) According to the passage, how is Elizabeth different than many of her classmates?**

A. She is diligent.

B. She is dishonest.

C. She is not wealthy.

D. She is anti-social.

**12) As used in the final paragraph, which is the best antonym for inevitable?**

A. unlikely

B. unmistakable

C. possibly

D. unclear

**13) Which of the following words best describes how Elizabeth feels after being accused of cheating?**

A. frustrated

B. removed

C. discouraged

D. enraged

**14) A character in a story who is opposed to, struggles against, or competes with another is called the antagonist. Using this information, it can be understood that the antagonist in this passage is**

A. Elizabeth

B. Mr. Billings

C. Elizabeth's accuser

D. the recommendation committee

**15) According to the passage, the Blauvelt Award is important to Elizabeth because it will**

A. demonstrate her natural intelligence

B. help her pay for college

C. make her parents proud

D. prove her innocence

**Candy Store**

Trudy, Emmett, and Carlo went to their favorite candy shop, The Candy Cabin. Each person brought his or her own money. At The Candy Cabin, the friends each purchased their favorite types of candy.

Trudy had $5.00 from her weekly allowance. Each week Trudy took out the trash, washed the dishes, and made her bed daily. For these chores, she received $5.00. This week she decided she was going to spend all of her money on candy bars. Trudy loved candy bars, so she purchased an Amazing Almond Bar, two Crazy Caramel Bars, and a Totally Choco-Monkey Bar.

Emmett had $2.00 that his mother had given him earlier

that morning. Emmett was allergic to nuts and chocolate, so he spent his money on Fruity Gummy Blasters. He did not buy many though. That's because he got a cavity in his tooth. His dentist told him to eat less sugar. For this reason, Emmett needed to restrict the amount of sugar he eats.

Carlo came to The Candy Cabin with $4.00. He wanted to purchase a Super Candy Sampler Pack, which he knew cost exactly $4.00. A Super Candy Sampler Pack included two candy bars, two packs of chewing gum, three types of Gummy Blasters, and a mystery treat. When Carlo reached into his pocket to get his money, he noticed that he only had $3.00. One of his dollars must have fallen out! Carlo looked all over the shop. But he did not see his dollar anywhere.

"Well, I guess I won't be getting the Super Candy Sampler Pack," he said. His face looked glum. Trudy reached into her pocket and pulled out a one-dollar bill. "Here you go," she said.

"Hey!" Carlo smiled. "Thanks!"

- 1. **16) Trudy wanted to spend all of her money on**
  2. A. Fruity Gummy Blasters
  3. B. candy bars
  4. C. nuts and chocolate
  5. D. a sampler pack

**17) Using the information in the passage as a guide, who is interested in trying different things?**

**I. Carlo**

**II. Emmett**

- 1. **III. Trudy**
  2. A. I only
  3. B. I and II only
  4. C. II and III only
  5. D. I, II, and III
  6. **18) In paragraph 3 the author writes, "For this reason, Emmett needed to restrict the amount of sugar he eats." Which of these people restricts something in the same way that Emmett does?**
  7. A. Marcus, who practiced his football skills all summer so he would be prepared for football tryouts.
  8. B. Nicholas, a baker, who tried many types of sugary treats at the new downtown bakery.
  9. C. Robin, who needed help unloading her car because she purchased a large amount of groceries at the supermarket.
  10. D. Marissa, who had to put her dog on a leash after he played too roughly with another dog at the park.
  11. **19) With the money Trudy gave him, Carlo will most likely**
  12. A. purchase a Super Candy Sampler Pack
  13. B. ask his mother for more money when he gets home
  14. C. buy an extra treat for Trudy and Emmett
  15. D. save his money so he has enough for next time
  16. **20) As used near the end of the passage, the word glum has its nearest OPPOSITE in**
  17. A. upset
  18. B. interested
  19. C. strong
  20. D. happy
  21. **21) Using the information in the passage, we can understand that**
  22. A. Food and water aren't always available in the desert.
  23. B. Camels can have up to three humps on their backs.
  24. C. Camel humps can weigh up to 100 pounds.
  25. D. Lots of animals have humps on their backs.
  26. **22) This passage mostly provides**
  27. A. information on how long animals can go without food and water
  28. B. facts about what food is available in the desert
  29. C. information about how much a camel's hump can weigh
  30. D. facts about camels and the humps on their backs

**Vacation Opinions**

*In this passage, two students give their opinions about vacations.*

Tandy’s Opinion

The beach is the best place to take a vacation. At the beach, there are many things to do. People can go swimming in the ocean, walk along the shore, or play in the sand. There are lots of kids to play with at the beach. The scenery there is also very beautiful. The brown sand and the crystal blue water are very pretty. Sometimes you can even see dolphins jumping on the horizon. It is also nice to

listen to waves crashing on the shore. That’s what is so great about the beach.

Dion’s Opinion

Going to the mountains is the best vacation. In the mountains, there are a lot of different activities that people can do. Going hiking, climbing, swimming, or telling stories around the campfire are just a few. The scenery in the mountains is far more beautiful than anywhere else. The tall mountains, the green trees, and the views are the best! In nature, you can see many types of plants and animals too. The mountains are also quiet. You can have time to be by yourself, because it is not very populated. It is more relaxing to take a vacation in a place like this. The mountains are the best place to go on vacation.

**23) Which of the following sentences best summarizes Tandy’s opinion?**

A. For its many activities and beautiful setting, the beach is the best place to go for a vacation.

B. Even though the mountains are very calm, the beach is a quiet place to visit.

C. The beach is the best place to go on vacation, because it is much warmer than anywhere else.

D. Because of the many different types of animals that you can see, the beach is the best place to vacation.

**24) Which of these reasons is/are used by BOTH Tandy and Dion to support their opinions?**

**I.** There are lots of kids to play with.

**II.** The landscape is nice.

**III.** There are many things to do.

A. I only

B. I and II only

C. II and III only

D. I, II, and III

**25) According to Tandy, what is pretty at the beach?**

**I.** brown sand

**II.** crystal blue water

**III.** colorful sunsets

A. I only

B. I and II only

C. II and III only

D. I, II, and III

**26) As used by Dion, the word populated most nearly means**

A. noisy

B. crowded

C. quiet

D. popular

**27) Tandy and Dion are most likely responding to which of these questions?**

A. Why would someone want to vacation in the mountains?

B. Where do you want to live when you grow up and why?

C. What is the best place to take a vacation and why?

D. Why is the beach the best place to take a vacation?

**Something Special**

Marco’s wife's birthday is in two days. Marco wants to do something special to celebrate. He decides to cook her a special birthday dinner. He thinks about what she would like to eat. Her favorite American food is hamburgers, so he is going to make hamburgers for her.

The first step for Marco is to make a grocery list. The list helps him remember to buy all that he needs for the meal when he goes to the store. Here is his grocery list:

*hamburger buns ketchup rice*

Marco asks his daughter, Sara, if she can think of anything else her mother would like to eat. Sara reminds her father that Mom also likes broccoli. He adds this to the list.

*Hamburger*

*buns*

*Ketchup*

*Rice*

*Broccoli*

"Look in the condiments aisle of the grocery store to find the ketchup," Sara tells her father.

At the store, Marco rolls the cart up and down the aisles. He looks at his grocery list. He buys hamburger, buns, ketchup, rice, and broccoli. In the frozen food section, he looks at the ice cream a long time. But he does not put any ice cream in his cart.

Instead, Marco goes to the bakery section of the store. He buys a pretty white cake with blue candles. Blue is his wife’s favorite color.

The man behind the bakery counter asks Marco, "Would you like me to put a name on the cake?" Marco nods his head and tells the baker his wife’s name.

The baker turns around and works on the cake. When he gives the cake to Marco, the cake says, "Happy Birthday Maria!"

Marco knows his wife will be surprised by her favorite foods and the special cake on her birthday.

**28)** Marco decides to celebrate his wife’s birthday by

**I. cooking dinner for her**

**II. buying a cake for her**

- 1. **III. having a party for her**
  2. A. I only
  3. B. I and II only
  4. C. II and III only
  5. D. I, II, and III
  6. **29) How does the reader know the name of Marco’s wife?**
  7. A. Sara talks to Marco about her.
  8. B. Marco writes her name on his grocery list.
  9. C. The baker writes her name on the cake.
  10. D. The reader never learns the name of Marco's wife.
  11. **30) How is Sara related to Maria?**
  12. A. Sara is Maria’s friend.
  13. B. Sara is Maria’s mother.
  14. C. Sara is Maria's sister.
  15. D. Sara is Maria’s daughter.
  16. **31) According to the passage, Marco does not buy the ice cream because he**
  17. A. does not see a flavor that he likes
  18. B. decides to buy a birthday cake instead
  19. C. is afraid it will melt before he gets home
  20. D. remembers that his wife does not like it
  21. **32) How does Sara help her father with dinner?**
  22. A. She lets him know that it is his wife’s birthday.
  23. B. She reminds him that her mother likes broccoli.
  24. C. She goes to the grocery store with him.
  25. D. She cooks the hamburgers for him.

**The Rent Man**

Someone is knocking on Amanda’s door. Amanda is home, but she does not answer. It is the man who owns the house where she lives. His name is Mr. Campbell. Amanda calls him the Rent Man. He has come by to get the rent money Amanda owes, but Amanda does not have the money to pay him. That's because Amanda lost her job at the auto factory three and a half weeks ago.

"I worked there for 15 years," Amanda thinks to herself. She is bitter. "But it took them just one day to take my job away."

Amanda has no idea when she will find another job. She looks for work most every day.

She looks for work at a restaurant. "You have never worked at a restaurant," the owner tells her. "Unfortunately, we are looking for someone with experience."

She looks for work at the bookstore. "We don’t have any jobs right now," the clerk tells Amanda.

She looks for work at the grocery store. "I will call you to let you know," the manager says. Amanda is worried. She is running out of money quickly.

There is another loud knock at the door. Amanda sits quietly in her kitchen, hoping the Rent Man will go away soon.

**33) According to the passage, when did Amanda lose her job?**

A. ten days ago

B. two weeks ago

C. two and a half weeks ago

D. three and a half weeks ago

**34) In paragraph 2, we learn that Amanda is bitter. This means she feels**

A. disappointed and angry

B. confident and happy

C. hungry and tired

D. quiet and alone

**35) According to the passage, where does Amanda look for a new job?**

**I.** a restaurant

**II.** a bookstore

**III.** a grocery store

A. I only

B. I and II only

C. II and III only

D. I, II, and III

**36) Based on information in the passage, we can understand that Amanda MIGHT get**

A. the restaurant job

B. the bookstore job

C. the grocery store job

D. none of the jobs

**37) It could have been helpful for Amanda to tell Mr. Campbell that**

A. she will call the police if he knocks again

B. he had better stop knocking on her door

C. she recently lost her job at the factory

D. she will have the rent for him tomorrow

**38) What would it be reasonable for Mr. Campbell to do next?**

**I.** break the door down

**II.** come back another day

**III.** call Amanda on the phone

A. I only

B. I and II only

C. II and III only

D. I, II, and III

This document and its content is protected under copyrights laws and owned solely by ReadTheory Limited

Partnership; Distributing, reusing, republishing the document in any way or form is forbidden.
